# ICU Nurses’ Views on the Potential Design and Anticipated Consequences of a Hypothetical Pay-for-Performance (P4P) Program in Greece: An Exploratory Qualitative Study

**DOI:** 10.7759/cureus.96308

**Published:** 2025-11-07

**Authors:** Dimitrios Kosmidis, Georgios Manomenidis, Chrysoula Zdagka, Dimitra Armeni, Stefanos Mavroudis, Sotiria Koutsouki

**Affiliations:** 1 Department of Nursing, Democritus University of Thrace, Alexandroupolis, GRC; 2 Department of Genomics, Erasmus University Rotterdam, Rotterdam, NLD; 3 Department of Nursing, University of Thessaly, Larissa, GRC; 4 Intensive Care Unit, General Hospital of Kavala, Kavala, GRC

**Keywords:** incentives, intensive care units, nursing, pay-for-performance, pre-implementation, qualitative research, remuneration

## Abstract

Background

In Greece, there is no pay-for-performance (P4P) program in ICUs. Evidence on ICU nurses’ pre-implementation attitudes toward remuneration and hypothetical P4P design is scarce.

Objective

To elicit ICU nurses’ hypothetical views on (a) the current remuneration system, (b) key design features of a potential ICU P4P scheme, and (c) anticipated consequences (benefits, risks, and unintended effects).

Methods

This is an exploratory qualitative study conducted using reflexive thematic analysis. ICU nurses meeting three eligibility criteria (bachelor’s degree in nursing, ≥ three years’ ICU experience, and written informed consent) were recruited via social media convenience sampling (June 2025). Nine nurses responded; seven were eligible and were interviewed via secure video conference (50-95 minutes; Greek). All participants worked in public hospitals across six Greek health regions. Two researchers independently double-coded all transcripts with third-party adjudication. A shared codebook, versioned analytic memos, and an audit trail supported rigor.

Results

Participants perceived pay as disproportionate to ICU intensity and workload and distinguished three facets of dissatisfaction: pay level (adequacy of the base salary), pay structure (seniority-graded pay misaligned with role/unit demands), and pay type (base-only salary lacking performance-linked elements). They expressed conditional openness to team-based P4P under safeguards: adequate staffing and information systems; a small set of clearly defined, locally feasible, nursing-sensitive indicators co-designed with the interprofessional ICU team (nurses and physicians); realistic, negotiated targets; transparent bonus criteria; and credible, transparent governance (independent auditing and clear accountability). Anticipated risks included gaming, crowding out, and interprofessional tension. Expected gains were modest and context-dependent rather than transformative.

Conclusions

Participants expressed conditional openness to team-based P4P only if specific safeguards are in place (adequate staffing and information systems; locally feasible, nursing-sensitive indicators co-designed with the ICU team; transparent governance and bonus criteria). These are baseline, pre-implementation attitudes from a small, self-selected, non-representative sample of ICU nurses with no P4P experience. They are subject to hypothetical response bias and the intention-behavior gap and should inform whether and how to pilot-test team-based incentives, rather than justify direct implementation or population-level claims.

## Introduction

High workload and poor quality of working life are well-documented pressures that erode nurses’ job satisfaction and effectiveness in intensive care units (ICUs), and evidence indicates that they also compromise the quality of care delivered [1]. These pressures should be explicitly considered when formulating new health policies, including pay-for-performance (P4P) programs [2]. P4P is an approach in which financial or other incentives are provided conditionally upon completing a specific action or meeting predefined performance targets. The model is directed primarily at healthcare professionals and organizations, rewarding the delivery of specified services, the provision of high-quality care, and/or the attainment of better patient outcomes [3]. P4P can be instituted at multiple tiers of a health system, i.e., at the individual level (clinicians), the organizational level (clinics and hospitals), and the administrative level (regional/local authorities, non-governmental organizations (NGOs), and municipalities).

In Greece, ICU nurses work with empathy and responsibility under complex technology and severe patient acuity. However, they also express frustration due to chronic staff shortages, which have led them to work at the limits of their capabilities [4]. Safe and timely care in ICUs depends on nurses’ comprehensive understanding of both patients’ clinical status and the unit’s administrative operations, policies, and workflows, which enable prompt and appropriate procedural responses. Therefore, any exploration of a potential P4P program should take these multifaceted requirements into account and incorporate meaningful participation by nurses themselves so that the program design aligns with their needs. Such initiatives should include both clinical nurses providing direct care in the ICU and nursing managers or leaders, as their participation in the formulation and evaluation of institutional P4P policies is important [5]. Historically, P4P programs have centered on physicians, with limited involvement of nurses and nursing care. Consequently, the potential impact of P4P on nursing work remains insufficiently understood [6]. Moreover, while early expectations for P4P were high, they have been tempered by the complexity and heterogeneity of implementation across clinical environments, underscoring the need to examine the nursing role more closely [7]. Given the absence of comparable studies in Greece and the limited knowledge base on this topic, we conducted a qualitative study to explore ICU nurses’ hypothetical views on the potential design of a P4P program in the Greek context.

In this study, we elicit ICU nurses’ hypothetical views on the potential design, acceptability, and anticipated consequences (intended and unintended) of a P4P program in Greek ICUs. Given the absence of P4P in the participating settings, our focus is pre‑implementation: baseline attitudes and the reasoning that underpins them, not evidence of implementation experience or effectiveness. The study was guided by the following research questions (RQ): RQ1: Under what safeguards and contextual conditions would ICU nurses consider a hypothetical P4P acceptable? RQ2: Which design features (e.g., indicator selection, co-design, measurement, and attribution; auditing/governance and trust; team- vs. individual-based incentives) do nurses prioritize, and why? RQ3: What anticipated benefits, risks, and unintended consequences do nurses foresee for teamwork, morale, equity, and care quality?

## Materials and methods

Study design

We conducted an exploratory qualitative study using reflexive thematic analysis to elicit ICU nurses’ hypothetical views on (a) the potential design features and (b) the anticipated consequences of a P4P program in Greek ICUs, where no P4P currently exists. This exploratory-descriptive orientation is appropriate for under-researched phenomena and for capturing participants’ subjective experiences and the influences shaping their perspectives [8-12]. We used semi-structured, open-ended interviews. We drew on the instrument developed by Meterko et al. [13], which is a validated 26-item survey designed to assess provider attitudes toward P4P programs, as a conceptual scaffold for our interview guide. The instrument operationalizes seven domains hypothesized to shape responses to P4P: awareness/understanding, clinical relevance, cooperation, control, financial salience, unintended consequences, and perceived impact (psychometric evidence reported by the authors; Cronbach’s α ≈ 0.50-0.80 across scales) [13]. We did not administer the questionnaire; instead, we converted its closed-ended items into open-ended prompts tailored to ICU nursing and team-based care (e.g., awareness/understanding of indicator definitions and feedback; control/cooperation regarding dependence on physician orders and unit resources; financial salience regarding perceived bonus magnitude and threshold setting). This approach ensured content coverage of salient P4P features while preserving an exploratory, pre-implementation focus appropriate to the Greek ICU context. The full interview guide is provided in the Appendices.

Participants and recruitment

We recruited ICU nurses via social media convenience sampling (ICU professional groups/pages) to elicit hypothetical views on potential P4P design and consequences. We used this approach due to the practical difficulty of accessing a geographically dispersed, highly specialized, and time-constrained ICU workforce for in-depth interviews. Recruitment took place in June 2025 via posts in ICU professional groups/pages on social media (no incentives). The invitation outlined the study purpose, interview duration (50-95 min), the main eligibility requirements (registered nurse with a bachelor’s degree in nursing, ≥ three years’ ICU experience, willingness to provide written informed consent, and access to videoconferencing), confidentiality/voluntariness, and contact details. It did not solicit pay complaints. We received nine responses; seven met eligibility and were interviewed; two were excluded for less than three years of ICU experience. The recruitment notice clarified that no P4P program currently exists. Given the channel and topic, self-selection bias is likely; the sample is small, self-selected, and non-representative. Given the salience of remuneration, volunteers may have been more motivated to voice pay-related concerns; the sample may therefore be enriched for dissatisfaction, and findings should be interpreted cautiously with respect to attitudes on pay. Eligibility was restricted to adult ICUs; pediatric ICUs and cardiac/coronary care units (CCUs) were not eligible (see Inclusion and Exclusion Criteria).

We do not claim full thematic saturation [14]. Instead, we documented data sufficiency for exploratory aims based on (i) independent double coding with adjudication and a shared codebook, (ii) an audit trail (decision memos/codebook revisions), and (iii) convergence of key safeguards/conditions across interviews. Given the small, self-selected sample, findings are not generalizable and are used for hypothesis generation only.

Inclusion and exclusion criteria

All types of adult ICUs (medical, surgical, mixed, and others) from both university and non-university hospitals were eligible. Pediatric ICUs and CCUs were excluded. Eligible participants were: (a) registered nurses with a bachelor’s degree in nursing; (b) at least three years of ICU clinical experience, to ensure sufficient exposure to day-to-day ICU practice, quality-of-care issues, staffing pressure, and remuneration concerns; and (c) those willing to provide written informed consent. Participants were also required to have access to a computer with an online communication platform for the video conference interview.

Data collection

Interviews were conducted in June 2025 at scheduled times. Each interview began with an explanation of the study’s purpose and the intended use of its findings. Semi-structured interviews followed an interview guide covering indicator selection, co-design, measurement, and attribution; auditing/governance and trust; team- vs. individual-based incentives; anticipated benefits/risks; and perceived impacts on care quality. Participants were reminded that the scenario was hypothetical and that no P4P program currently exists. Interviews were conducted via secure videoconferencing, in Greek; English quotations were forward-translated by a bilingual researcher and cross-checked by a second bilingual team member to preserve meaning and tone. No incentives were offered.

Ethical considerations

Participation was voluntary, and written informed consent was obtained via email prior to the interviews. We acknowledge that the recruitment mode may have influenced those who volunteered (self-selection). Participants were assured of confidentiality and informed about measures to protect their identity, including removal of identifiable information from transcripts [15]. To maintain anonymity, participants were assigned codes (N1-N7). They were informed of their right to withdraw at any time without consequence. Ethical approval was granted by the Research Ethics Committee of Democritus University of Thrace (Approval No. 62744/531; dated: 22/5/2025).

Analysis and rigor

All interviews were transcribed verbatim. We conducted a reflexive thematic analysis following Braun and Clarke’s six-phase approach, as summarized by Nowell et al. [16]. In our application, we did not treat “defining and naming themes” as a standalone phase; instead, theme definition and naming were integrated into theme review and report writing. Accordingly, the enacted phases were familiarization with the data, generation of initial codes, search for candidate themes, review and refinement of themes, and report production. We did not claim full saturation; rather, we established analytic sufficiency for exploratory aims through an audit trail, iterative codebook revision, and convergence of interpretations across coders. Illustrative quotes in the Results are attributed to participant ID (N1-N7).

To enhance trustworthiness and dependability, two researchers independently coded each transcript and resolved discrepancies through discussion, with a third reviewer adjudicating any outstanding differences. Coding was conducted in a shared digital workspace with a maintained codebook and versioned analytic memos (audit trail). The team held regular peer-debriefing meetings, and comparative analysis examined convergence across ICUs and demographic profiles [17]. Member checking was not feasible; instead, we relied on double coding, adjudication, and documentation of analytic decisions to enhance credibility. Identifiers were removed during transcription, and all materials were stored on a password-protected drive.

## Results

Sample and interview corpus

During June 2025, nine ICU nurses responded to our social media call for participation. Seven met the eligibility criteria (registered nurse with a bachelor’s degree, ≥ three years’ ICU experience, and provision of informed consent) and completed an interview (50-95 minutes, in Greek). As recruitment relied on social media and yielded a small response, this is a self-selected, non-representative sample; we therefore refrain from population-level claims and present hypothesis-generating insights only. One nurse was employed in a university ICU, five held a master’s degree, and one was a doctoral candidate. ICU experience ranged from four to 16 years, while total professional experience ranged from 10 to 25 years. All participants worked in public healthcare facilities, with three also having prior experience in the private sector. Detailed demographic characteristics are presented in the Appendices. Thematic analysis identified seven themes: (1) perceived salary inequities and pay disparities; (2) lack of financial incentives and dissatisfaction with the existing pay system; (3) anticipated benefits under a hypothetical P4P; (4) design considerations for a hypothetical P4P; (5) trust, governance, and stakeholder participation; (6) perceived impact on quality - minimal change expectations (counterpoint); and (7) team dynamics and potential conflict. Table 1 summarizes the research questions, themes, subthemes, and illustrative quotes.

**Table 1:** Research questions, themes, subthemes, and illustrative quotes from a small, self-selected sample of ICU nurses in Greece. Findings reflect hypothetical attitudes from a small, self-selected, non-representative sample; not generalizable. Quotes labelled by interview ID (N1–N7). P4P: pay-for-performance.

Research questions (RQ)	Themes	Subthemes	Illustrative quote
RQ1/RQ3	1. Perceived salary inequities and pay disparities	1.1 Inequities in financial compensation	N1: “The pay’s way lower than what ICU nurses really deserve.” N2: “Pay’s the same for everyone (based on years on the job and your degree) … and I think it’s not enough.”
				1.2 Pay disparities compared to other departments	N3: “…I think every unit has its own quirks, but for some I know well, [the pay] should definitely be higher”. N4: “Pay—especially in hospitals—should match how intense the unit you work in is… not all of us getting the same pay. In the ICU, it should also reflect the extra workload compared to other units.”
		RQ3	2. Lack of financial incentives and dissatisfaction with the existing pay system	Reduced discretionary effort/ “minimum effort”	N1: “You see people skipping certain care because, you know, we all get paid the same… Why should I do extra? … So you just do the bare minimum”. N6: “Sometimes, when I think about how we’re paid, I get discouraged… and yeah, sometimes my performance drops.”
RQ3	3. Anticipated benefits under a hypothetical P4P	3.1 Income increase	N1: “It’d definitely be a chance to raise my income, which, I’ll say again, is unacceptable as it is, since it pays me the same as everyone else.”
		3.2 Perceived potential improvement in patient care	N4: “I don’t think there’d be a big change… but there would be some improvement.” N5: “Yeah, I think so—if nurses had those extra incentives, they’d perform better… we’d see better results in the ICU.”
RQ2	4. Design considerations for a hypothetical P4P	4.1 Targets/indicators (selection & attribution)	N1: “…infections—especially the ones tied to central venous catheters” N2: “Definitely infections… pressure ulcers… and family/caregiver satisfaction” N3: “…infections and ventilator-associated pneumonia (VAP), plus medication-administration errors” N4: “…I’d say infections… are a key piece on their own… medication administration too… and falls could be tied in as well” N5: “…patient care to prevent VAP… patient falls—even if they’re rare—are obviously linked to nursing care… pressure ulcers, VAP, and infections would probably be the three biggest indicators” N7: “I’d say the most important ones are infections, patient falls, medication [administration] errors, pressure ulcers, and family satisfaction.”
		4.2 Measurement challenges & external influences	N1: “…I don’t agree with using bed occupancy—because you can have high occupancy just because some ICUs are doing their job well… and a patient can still fall if they’re super agitated, or if the staffing ratio is off.” N4: “…but for anyone to measure these things, they’ve got to be reported. I might make a mistake and just not say it… or even hide it.” N5: “ICU length of stay isn’t just on nurses—at least not solely. It depends on patient acuity, the doctors, and so on.” N3: “…the problem here is time—there might not be enough time for a nurse to give the care needed to hit a complex goal.”
		4.3 Size and structure of incentives	N1: “I don’t think it has to be a huge raise… Like, I’m getting about €1,300 right now—if they added even €200, that would, at least to a degree, reflect my effort.” N1: “I’d probably rather have the whole pay be performance-based… or have a low base pay and then add a bonus—and a penalty—on top.” N4 (1): “I think there should be a standard base salary plus a bonus for hitting certain goals. Docking pay—penalties—wouldn’t be a good system for workers.” N4 (2): “It doesn’t have to be huge… ideally, something like a 20% bonus or extra pay.”
		4.4 Team- vs. individual-based incentives	N1 (1): “…I’d go with mixed metrics, because in the ICU you can’t really say something is purely ‘nursing’; it’s not that clear what your own share of responsibility is. To start with—since we’re talking first steps—I’d rather make them team-based… as the ICU staff as a whole. I think incentives make more sense at the unit level than as something personal.” N1 (2): “…but since ICU work is team-based, you could still assign a nursing share—like 50–50 with the doctors, or 80–20.” N4: “I think individual targets are unrealistic because we work in shifts… the patient doesn’t depend only on us; our work is about collaboration and unity. It should be team-based, not individualized.” N5: “This is collective work—teamwork, not solo. In an ideal world the reward would be personal, but in the ICU that’s not feasible; it has to be at the team level. It could even be shared with the doctors—they provide a different kind of care in the ICU, nurses have their own role, but there’s a link… a common goal along the patient’s course. Quality goals should be shared—like cutting down infections.” N6: “[The goals] should be as shared as possible.”
		4.5 Determining and negotiating targets	N1 (1): “Goals need to be clearly spelled out… and there should be room to negotiate a few extra things…” N1 (2): “As long as there’s a financial incentive for some people… if certain metrics were left outside the incentives, personally—yeah—I wouldn’t treat them any differently.” N4: “Which goals get set—mainly (answering tο Q10)—could be negotiable, but again, it depends on the goal.” N5: “It’d be good to have some room for negotiation.”
RQ2/RQ3	5. Trust, governance, and stakeholder participation		N1 (1): “…if you choose a metric where the results are the worst, that’s a challenge in itself—it could lead to takeaways or to improving that metric. I’m guessing when a process like this rolls out, they’ll bring in the right people to pick the metrics. If the folks setting them are competent and know the field, then yeah. I can’t say it with certainty—it does worry me a bit—but I believe it’ll be done properly. And if the selection’s done by people from the ICU world, I don’t think you can really go wrong.” N4 (1): “Yeah, I’d be concerned about who’s involved in picking the metrics—whether they’re responsible and people I can trust.” N1 (2): “Trust the administration/the people in charge or the policymakers to implement something like this in the ICU? No. I’d have more confidence if the health professionals were coming out of ICU practice.” N4 (2): “I wouldn’t trust them. Honestly, I’m disappointed with the current government and don’t trust it overall… ICU people should be more involved in organizing a program like this.”
RQ3	6. Perceived impact on quality — minimal change expectations (counterpoint)	Minimal change expectations	N1: “As for neglecting some patients’ care, I think an ICU that didn’t roll out a program like this would probably be worse.” N2: “…I’d maybe be a bit more careful—more focused than before… yeah, there’d be a tiny difference, but neglect other patients? No.” N4: “We’re careful even without extra pay… I’d be just as focused on a specific goal even without financial incentives.” N6: “No, I don’t think there’d be neglect… with the way we’re paid now, I don’t think some patients are being neglected. Maybe there’d be a very small difference.”
RQ3	7. Team dynamics and potential conflict		N1: “At work, with that same logic, we already see clashes—‘we all get paid the same, so why should I do it?’—that’s a form of conflict too”/ N7: “I think the conflicts would mostly be between nurses, and less between doctors and nurses.”

Analysis of results

Theme 1: Perceived Salary Inequities and Pay Disparities

We use “injustices” to denote objections to the pay architecture itself (e.g., seniority-based pay misaligned with job demands), and “inequalities” to denote pay disparities relative to workload, responsibilities, or unit intensity. Given that interviews explicitly probed remuneration and recruitment was convenience-based via social media, topic salience and self-selection likely enriched the sample for pay dissatisfaction. The findings, therefore, describe perceptions among volunteers who engaged with a remuneration-focused interview and do not estimate the prevalence of dissatisfaction among ICU nurses in Greece. To avoid conflation, we distinguish three facets of dissatisfaction: pay level (adequacy of the base salary), pay structure (seniority-graded pay misaligned with role demands or unit intensity), and pay type (a base-only salary lacking performance-linked elements). Quotes were coded accordingly.

Inequities in financial compensation (level): Participants emphasized that wages were lower than what ICU nurses truly deserved, particularly considering the workload and responsibilities. As N1 stated: “…the salary is much lower than what ICU nurses actually deserve… because in the ICU the workload is very high.” Dissatisfaction was also expressed with a compensation system based primarily on years of service rather than job demands.

Pay disparities compared to other departments (structure): Participants argued for differentiated pay according to the intensity and demands of each unit. N4 noted: “Salaries, especially in the hospital, should reflect the workload of the department you work in… and not everyone receiving the same amount.”

Theme 2: Lack of Financial Incentives and Dissatisfaction With the Existing Pay System

Building on the three remuneration dimensions described in Theme 1 (pay level, pay structure, and pay type), participants also described how the absence of meaningful financial incentives contributes to frustration and reduced discretionary effort. Participants recognized that the lack of financial incentives and the resulting frustration could diminish performance. As N1 observed: “You see behaviors where someone skips certain care tasks because we all get paid the same… Why should I do more?… So you end up doing the minimum.” We coded this statement primarily to pay structure (equal pay irrespective of differential effort/workload), with secondary coding to pay level, where participants emphasized low wages per se. The pay type dimension is elaborated where participants discuss preferences for team-based bonuses under a hypothetical scheme (see Theme 4).

Theme 3: Anticipated Benefits Under a Hypothetical P4P

Following the interview guide, questions probed expectations under a hypothetical scheme linking quality targets to financial incentives in ICUs. This theme was also linked to perceived problems with the current pay system, as expected benefits combined potential financial gains with perceived improvements in patient care.

Income increase: The opportunity to increase income through incentive-based programs was appealing. N1 stated: “It would definitely be a chance to increase my income, which, I must say again, is unacceptable as it equates me with everyone else.”

Perceived potential improvement in patient care: Participants perceived financial incentives as both a fairer compensation mechanism and an opportunity to enhance patient care. N4 explained: “I don’t think there would be a major difference [in care]… but there would definitely be an improvement… we would also see an impact on quality.” Similarly, N5 added: “If nurses had these additional incentives, they would perform better… we would see better outcomes in the ICU” (anticipated effects rather than measured outcomes).

Theme 4: Design Considerations for a Hypothetical P4P

Participants were asked to comment on examples of quality and efficiency measures that could be linked to financial incentives in intensive care (e.g., infection rates, medication errors, and patient falls) and to suggest additional indicators for a hypothetical P4P program in the ICU. While agreeing with some indicators, they also highlighted difficulties regarding the choice, measurement, and implementation of such targets.

Targets/indicators (selection & attribution): Participants mentioned quality/efficiency indicators connected with nursing care and discussed their validity. They agreed with some but expressed skepticism about others. “ICU length of stay doesn’t depend only on nurses… at least not exclusively… it depends on illness severity, on the physicians, etc.” (N5).

Measurement challenges & external influences: Despite agreement on some indicators (e.g., ventilator-associated pneumonia, medication errors, pressure ulcers, and family satisfaction), concerns were raised about how these could be measured and external factors influencing them. N1 remarked that staffing could affect quality targets: “…I disagree with occupancy rate as an indicator… occupancy may be high because some ICUs do their job well… it may actually reflect good performance.” Regarding medication errors, N4 explained: “…but for someone to measure them, they must be reported. I may make an error but not disclose it… or even hide it.” Lack of time was also identified as a barrier. N3 noted: “…there may not be sufficient time for a nurse to provide the care required to achieve a complex, multi-indicator target.”

Size and structure of financial incentives: Views varied; many participants favored a stable base salary supplemented by bonuses for achieving certain quality/performance targets. Penalties (e.g., salary or bonus reductions) were generally rejected as potentially harmful to morale. N4 commented: “I believe there should be a stable salary and an additional bonus for meeting certain targets. A punitive system would not be good… Not necessarily a very large bonus… ideally, something like 20% extra compensation.”

Team vs. individual-based incentives: Participants emphasized that quality/effectiveness indicators cannot be attributed solely to nurses but require collective responsibility. Furthermore, it emerged from the discussion that other external factors (e.g., nurse-to-patient ratio and medical decisions) may be of great importance and may influence the results. As N5 stated: “The work is collective, it’s teamwork… not individual… in the ICU, incentives must necessarily be group-based… For example, reducing infections should be a shared goal.” Accordingly, participants generally preferred group-based rather than individual incentives, reflecting the collaborative nature of ICU work and challenges in attributing individual performance. Some suggested that participation be universal; views varied regarding bonus size and the number of targets met. N4 added: “I think individual goals are unrealistic because we work shifts… The patient does not depend solely on us. Our work is collaborative… [financial incentives] should be group-based, not individual.”

Determining and negotiating targets: According to participants, defining the specific quality targets was considered more important than the amount of financial incentives. N4 emphasized: “The most important thing is which targets are set…” and added, “…they could be negotiable, depending on the target.” Most agreed that targets should be clearly defined and agreed upon in advance, though with limited flexibility for negotiation as a scheme evolves. N5 noted: “It would be good if there were room for negotiation.”

Theme 5: Trust, Governance, and Stakeholder Participation

Participants expressed moderate to strong distrust toward institutions’ ability to design and oversee a credible P4P scheme. N1 stated: “…Should I trust the administration or policymakers to implement such a program in the ICU? No. If these decision-makers came from an ICU background, I would have more trust…”

Theme 6: Perceived Impact on Quality - Minimal Change Expectations (Counterpoint)

Several participants anticipated little to no change in care quality under a hypothetical scheme (e.g., N2, N4, and N6). N4 noted, “We’re careful even without extra pay… I’d be just as focused…” One participant suggested a slight improvement relative to settings without such programs (N1).

Theme 7: Team Dynamics and Potential Conflict

N1 acknowledged that conflicts among staff might increase due to competition: “In the workplace… even now there are conflicts over pay… in the sense that since we are all paid the same, why should I do more… that in itself is a kind of conflict”. N4, however, believed conflicts were not entirely dependent on the program’s implementation, noting: “…it has more to do with the human factor and each person’s character.” It is important to note that the findings reflect the views of participants in this small, self-selected, non-representative sample and are not generalizable; concerns are presented as concerns rather than as observed outcomes.

## Discussion

Salary injustices and inequalities in the ICU (Theme 1)

Participants perceived nurses’ pay as disproportionate to the acuity, workload, and demanding nature of ICU nursing under conditions of chronic understaffing, circumstances that diminish performance [18] and fuel staff conflict [19]. At the macro level, uniform pay schemes have been widely criticized internationally, consistent with participants’ reports that the existing incentive framework can even suppress nurses’ intrinsic motivation to provide the best possible care [20].

Inadequate remuneration has contributed to widespread burnout, high attrition, and the migration of already scarce skilled professionals to better-paying settings abroad [21]. Current reimbursement arrangements in many European countries and the United States rely on diagnosis-related groups (DRGs), which do not directly link the value of nursing care to nurses’ pay [22]. Proposed alternatives include explicit billing for nursing, either time-based or activity-based, using new Current Procedural Terminology (CPT) codes [23] or a shift toward value-based and P4P models that reward not only volume but also the effectiveness of nursing care [24].

Across Europe, activity-based funding has been implemented in many countries [25], yet ICU reimbursement systems still vary substantially [26]. Recent literature further reveals complex correlations among job satisfaction, compensation, working conditions, leaving the profession, and performance among ICU nurses [27-30].

Lack of financial incentives and dissatisfaction with the existing pay system (Theme 2)

Linking financial incentives to clearly defined quality/performance metrics can improve the care delivered to patients by shaping routine nursing behavior [31]. On the other hand, when rewards are absent or minimal, implicit norms and workarounds may lead to omissions of basic care [32].

Barriers to patient-centered nursing in ICUs include organizational constraints and the high-intensity therapeutic environment [33]. Prior studies also warn that poorly designed P4P schemes can increase workload and accountability without improving working conditions, risking frustration and gaming/manipulation of metrics [34].

In Greek ICUs, moderate-to-high burnout and lower job satisfaction compared with other specialties have been reported [35]. Reasons include understaffing and heavy workload. These factors can affect care quality and highlight the deep crisis in Greek nursing and the urgent need for changes in key policies. This context may explain why participants argued that measures must be carefully designed to recognize additional effort without eroding professional values.

Taken together, participants’ pre-implementation views point to hypothesis-generating considerations for a potential pilot team-based incentives program. These considerations include indicator selection/co-design/measurement/attribution, transparent governance and auditing, and team-based (rather than purely individual) incentives; perceptions of bonus magnitude also vary, with prior reports noting order-of-magnitude levels around ~15-20% being viewed as more meaningful than ≤5%.

Anticipated benefits of a P4P program (Theme 3)

Benefits for Health Professionals

P4P programs have been reported to offer financial and professional benefits in some contexts, though evidence is mixed across designs and settings. Through bonus payments, nurses can increase their income and strengthen their position within the health system [36]. Involving nurses in the design and evaluation of these programs further promotes professional recognition and enhances their bargaining power [37].

P4P systems may also improve working conditions by supporting education, increasing job satisfaction, and reducing burnout. Non-monetary incentives, such as flexible scheduling or additional leave, can likewise make these programs more attractive. That said, real-world implementation has often been limited: in many cases, the financial awards are small (2-4% of total pay), and insufficient adjustment for the complexity of nursing work or for patient acuity risks inequities and unrealistic expectations [36,37]. Moreover, because intensive care is inherently multidisciplinary, it has been argued that P4P should reward the entire team to avoid inequities and to foster collective performance [38].

Benefits for Patients

It has been proposed that, when carefully designed, incentive programs implemented as P4P may act as levers to improve patient-experience measures and overall patient-perceived quality; however, reported effects vary by design and context. In addition, comparison and recognition mechanisms appear to drive constructive performance improvements [39,40]. In ICUs specifically, Khanduja et al. [41] highlight benefits such as enhanced patient safety through reductions in iatrogenic complications, a focus on processes and interventions with proven effectiveness, stronger interprofessional collaboration, and greater patient trust in the system.

Despite a sizable body of studies reporting positive effects on quality [42], evidence for P4P’s impact on patient outcomes and population health remains mixed. This is due largely to heterogeneity in program design, underlying payment systems, and care settings [43,44]. All the above potential benefits require methodologically robust evaluations to be confirmed in the intensive care context.

Participants viewed P4P as both a potential source of financial support and a source of moral reward and recognition for their work, if incentives were based on teamwork and distributed transparently. At the patient level, they predicted small but significant improvements in processes and outcomes (e.g., infections, medication errors, pressure injuries, and family satisfaction) through stricter adherence to protocols and more time devoted to patients.

Indicator choice and measurement feasibility (Theme 4, subthemes 4.1-4.2)

Participants agreed that selected nursing-sensitive indicators could be linked to additional financial incentives in intensive care. In the ICU context, proposed indicators include mortality, length of stay, infections (e.g., central line-associated bloodstream infections and ventilator-associated pneumonia), unplanned extubations, readmissions, patient falls, medication administration errors, and pressure injuries [45-47]. Under the Donabedian model, many of these are outcome variables, whereas others, such as skill mix, staffing levels, education level, and the work environment, are structure variables [48,49]. Recent literature also points to additional indicators (oral hygiene, patients’ anxiety levels, sleep quality), which remain largely theoretically motivated and insufficiently evidenced [50].

Participants’ views on selecting and implementing nursing indicators/performance targets aligned with challenges reported internationally. These include lack of measurement standardization, inconsistent results, geographic concentration of studies (mainly in the US), imbalance in the attention given to different categories of indicators, and methodological heterogeneity [51]. Studies on specific indicators, such as medication errors, have highlighted additional problems such as a lack of commonly accepted definitions, user-unfriendly reporting systems, and limited specialized knowledge among nurses [52,53].

A further issue is that, although Donabedian’s structure-process-outcome framework is conceptually clear, in practice, some indicators are difficult to classify neatly as outcome or structure. An excessive emphasis on structure or outcome indicators weakens the link to the quality of nursing care itself. The selection of a set of indicators often tends toward what is easiest to measure, with the result that the indicators selected rarely reflect the basic principles and professional values of nursing care [54].

While existing indicator datasets are a useful starting point, several prerequisites, such as adequate information infrastructure, feasibility testing, local adaptation, and participatory team feedback, are recommended as necessary [47,55]. Finally, there is a need to involve clinical professionals in the selection of indicators/targets, enable collective discussion and reflection, and support continuous redefinition of goals (rather than adherence to rigid specifications) to avoid fixation on measurements [56].

Incentive design: Size, level, and target setting (Theme 4, subthemes 4.3-4.5)

Incentive size, reward structure, and whether payments are targeted to teams or individuals can influence program outcomes [57]. Team-based P4P is considered superior to individual schemes primarily because it strengthens cohesion and trust, promotes collaboration over competition, and fosters a sense of collective responsibility [58]. Especially in environments such as ICUs, individual appraisal may be perceived as unfair and can lower morale, discourage continued participation, and lead to quiet quitting [59].

Participants’ views on the size and form of financial incentives varied but converged on several points: the additional incentive needs to be meaningful [60] but not necessarily excessively large. There was also a prevailing view that a stable base salary could be combined with non-punitive bonuses for achieving specific quality targets. These views align with prior work suggesting that order-of-magnitude bonuses around ~15-20% are perceived as more meaningful, whereas ≤5% is often perceived as insufficient [31].

According to participants, the success of a P4P program depends mostly on clearly defined and auditable criteria and roles, so that staff know in advance what is expected of them. In parallel, targets should be predefined yet revisable through a documented negotiation process involving the ICU team and management, with explicit risk adjustment (e.g., case mix and staffing), regular review cycles, and recording/justification of any interim changes [61].

Lack of trust, governance, and stakeholder participation (Theme 5)

A common conclusion from participants’ responses is the need to involve professionals with sufficient experience. Although this has been highlighted in prior literature [62], it is particularly important in critical care, as ICU professionals know best both the indicators and the unit’s overall operations. Their involvement should encompass the design and organization of the program, with strong leadership by ICU management and meaningful professional involvement, and with careful scrutiny of external involvement to ensure credibility and relevance. Across interviews, a strong distrust was expressed toward senior hospital management and policy decision-makers (government) regarding their ability to design and oversee such a scheme. Despite these concerns, nurses hope for recognition and merit-based reward for their work. They also believe that clear procedures, together with necessary technical clarifications, will help prevent future misunderstandings [63]. To strengthen trust, participants proposed transparent data flows, independent auditing, and an appeals/reporting mechanism throughout implementation.

Perceived impact on quality and team dynamics and potential conflict (Themes 6-7)

Overall, participants highlight two dimensions: (a) unintended consequences - impact on quality of care and (b) team dynamics and potential conflict.

Unintended Consequences - Risks to Quality of Care

Several participants anticipated little to no change in care quality under a hypothetical P4P scheme (see Theme 6), tempering assumptions of large effects. Where potential quality risks are discussed in prior work, they are generally attributed to scheme design and measurement effects (e.g., indicator selection and attribution, reporting burden and under-reporting, and gaming/documentation artefacts), rather than to a systematic neglect of care in the absence of incentives. In our data, such concerns were framed as possibilities rather than observations and should be interpreted as pre-implementation perceptions, not measured outcomes. Some prior work suggests that strong professional values, perceived fairness, and clinician ownership of performance indicators may help limit unintended adverse incentives in P4P arrangements; however, these safeguards are highly context-dependent and cannot be assumed [61].

Team Dynamics and Potential Conflict

As for staff relations, the introduction of incentives may create new ethical dilemmas and workplace frictions due to potential competition [60]. The concerns expressed about possible conflicts are viewed as stemming from differences in roles and remuneration among nurses and are consistent with the literature [64]. Conflicts could also arise from some nurses who believe that financial incentives may jeopardize professional ethics and patient autonomy [65]. Disputes may likewise emerge over bonus distribution, especially if the process lacks transparency or is perceived to favor certain roles, individuals, or groups [64,66], while the size of the bonus may even be perceived as “insulting” if it is small [65]. An additional important source of conflict identified is the lack of information: when nurses remain uninformed or receive ambiguous information about program rules, confusion, disengagement, and potential conflicts over expectations and responsibilities may ensue [67].

Clinical and policy implications

Given the small, self-selected, non-representative sample and the pre-implementation focus, the points below are hypothesis-generating and purely speculative; they are intended to inform pilot testing rather than prescribe implementation. If a P4P pilot were to be explored in selected Greek ICUs, participants’ pre-implementation views suggest design considerations to be tested: (i) adequate staffing and information systems; (ii) a small set of clearly defined, nursing-sensitive indicators co-designed with the interprofessional ICU team (nurses and physicians), with transparent attribution and risk adjustment; (iii) team-based incentives, preserving a guaranteed base salary and testing achievement-based bonuses; and (iv) transparent governance, independent auditing, an appeals mechanism, and regular feedback cycles. Some participants (and prior work) referenced order-of-magnitude bonus levels (~15-20%), but views varied; any thresholds should be treated as illustrative and require feasibility testing and formal evaluation. Physician acceptability and cross-professional effects were not assessed in this study and should be evaluated in parallel before any broader rollout. These considerations reflect stated, hypothetical preferences from a small, self-selected group of ICU nurses with no direct P4P experience; such stated preferences may not translate into actual behavior or outcomes. They therefore require feasibility, acceptability, and impact evaluation under real-world conditions before any policy relevance can be inferred.

Limitations

This study has several limitations. First, only seven nurses from six Greek health regions were included. We did not claim full saturation; rather, we established analytic sufficiency for exploratory aims. The sample is small, self-selected, and non-representative, limiting transferability; a larger sample could yield different or more nuanced findings. Recruitment relied on social media convenience sampling on a remuneration-focused topic, which likely introduced self-selection bias and may have enriched the sample for nurses who were already motivated to discuss pay-related dissatisfaction.

Second, as a qualitative study based on interviews, the results may reflect subjectivity on the part of both participants and the research team during data analysis. Finally, a fundamental limitation is that all findings derive from stated, hypothetical preferences regarding a non-existent P4P scheme, provided by seven self-selected ICU nurses with no prior P4P experience in Greece. As such, the data reflect pre-implementation attitudes, not observed behavior or outcomes; they are susceptible to hypothetical-response bias [68] and the well-known intention-behavior gap [69]. Consequently, these narratives do not estimate the prevalence of any attitude among ICU nurses, do not predict actual responses to real incentives, and offer only minimal guidance for operational roll-out absent field testing. The findings should therefore be treated as hypothesis-generating. Future research should include the following: (i) larger, multi-stakeholder samples (including physicians and managers); (ii) incentivized (real-stakes) choice designs and/or small-scale field pilots; (iii) objective performance measures and data triangulation; and (iv) prospective evaluation of feasibility, acceptability, and unintended effects under real-world constraints.

## Conclusions

Participants perceived that pay is disproportionate relative to ICU intensity and workload in Greece, reinforcing nurses’ dissatisfaction with uniform pay models. Within this context, participants expressed conditional optimism about team-based P4P, anticipating at most modest, context-dependent gains when staffing is adequate and indicators are co-designed and feasible. These findings represent baseline, pre-implementation attitudes from a small, self-selected sample of ICU nurses, and they should inform, but cannot direct, future program design. Their appropriate use is to guide whether and how to pilot-test team-based incentives, with rigorous feasibility and impact evaluation.

## Appendices

**Table 2:** Demographic characteristics of the participants. F/M: female/male; U/NU: university/non-university. Health district: 1st = Attica, 2nd = Piraeus and Aegean, 3rd = Macedonia, 4th = Macedonia-Thrace, 5th = Thessaly & Central Greece, 7th = Crete.

ID	Gender	Age (years)	Educational level	Public/private hospital	Previews experience in the private sector	ICU experience (years)	Total experience (years)	ICU type	Health district
N1	F	48	MSc	Public	No	16	17	U	1st
N2	F	45	MSc	Public	Yes	7	15	NU	2nd
N3	F	43	BSc	Public	No	10	13	NU	3rd
N4	F	42	MSc	Public	Yes	6	16	NU	4th
N5	F	34	MSc	Public	Yes	4	10	NU	4th
N6	F	40	BSc	Public	No	5	14	NU	5th
N7	M	47	MSc, PhD (c)	Public	No	16	25	NU	7th

Interview guide

Participant code: ____________________ Interview date: ____________________

Initial and additional supplementary/clarifying interview questions:

1. What is your view of ICU nurses’ pay in Greece?

- What is your view of ICU nurses’ pay in Greece relative to workload?

- What is your view of ICU nurses’ pay in Greece relative to other care departments?

2. Do you have experience with programs that link performance or quality indicators/targets to additional financial incentives?

3. What is your view on establishing indicators/targets that are linked to financial incentives (e.g., additional pay) in the ICU?

- With an additional financial incentive, could you invest more time and effort in ICU patient care?

- Would a program of additional financial incentives be an opportunity to increase your income substantially?

- Could a financial incentive improve your practice behavior in the ICU?

- Do you believe that, by focusing on certain indicators/targets, your patients could receive better care?

4. Are you aware of indicators/targets that could be set and linked to additional financial incentives in intensive care?

- Which of the following ICU indicators/targets do you believe could be linked to additional financial incentives (list): mortality; length of stay in the ICU and in the hospital; infections and transmission of pathogens (e.g., central line-associated bloodstream infections); duration of mechanical ventilation; ventilator-associated pneumonia; unplanned extubations and re-intubations; patient falls; medication-administration errors; pressure ulcers; family/caregiver satisfaction.

- Which of the above indicators/targets best reflect the performance or quality of nursing care in the ICU?

- Can you mention other ICU indicators/targets that reflect the performance or quality of nursing care?

5. What would be, for you, a satisfactory size of the financial incentive (as a percentage of your current salary)?

- How important is the size of the additional financial incentive to you?

- How large would a financial incentive need to be to compensate for your additional effort and time?

6. In a program linking indicators/targets to financial incentives, would you prefer: your entire salary to depend on it; an additional payment (bonus) on top of a fixed base salary; or could it also include a penalty (reduction of bonus or salary)?

7. Should financial incentives in intensive care be linked to purely nursing indicators/targets, or to joint (nursing and medical), and why?

- Should the incentives in intensive care be team-based or individual?

- Should a program linking indicators/targets to incentives be voluntary or mandatory for all ICU staff?

- Should the size of the additional incentive relate to the degree of indicators/targets attainment, the number of indicators/targets achieved, or both?

8. In a financial-incentive program based on indicators/targets, what matters more to you: the indicators/targets or the size of the additional financial incentives?

9. In a financial-incentive program based on indicators/targets, should the mechanism linking indicators/targets to incentives be clearly defined and agreed upon in advance, or should it allow for negotiation during implementation?

10. In a program linking indicators/targets to financial incentives, to what extent would you be concerned about…

...the mechanism for selecting indicators or indicators/targets (and the criteria used)?

...the method for calculating financial incentives?

...the potential neglect of some patients in favor of improving care for others?

...the focus only on aspects of care tied to the indicators/targets?

11. Do you believe you would be equally focused on care-based indicators/targets even without additional financial incentives?

12. To what extent would you trust the design and implementation of a program linking indicators/targets to financial incentives in the ICU by…

...the hospital administration/organizational leaders or government policymakers?

...people with ICU experience (physicians and nurses)?

13. Do you believe that a program linking indicators/targets to financial incentives…

...might lead to ethical dilemmas?

...might lead to conflicts among staff (nurse-to-nurse, nurse-physician, etc.)?

...would foster healthy competition to the benefit of the patient?

14. What would your overall stance be (generally positive or generally negative) toward creating and implementing a program that links indicators/targets to financial incentives in the ICU in the near future?
